# Cohesin loading factor Nipbl localizes to chromosome axes during mammalian meiotic prophase

**DOI:** 10.1186/1747-1028-8-12

**Published:** 2013-08-22

**Authors:** Katarzyna Kuleszewicz, Xiangwei Fu, Nobuaki R Kudo

**Affiliations:** 1 IRDB, Department of Surgery and Cancer, Imperial College London, Hammersmith Hospital, London W12 0NN, UK; 2 Present address: College of Animal Science and Technology, China Agricultural University, Beijing 100193, P.R. China

**Keywords:** Cohesin, Cohesin loading factor, Nipbl, Scc2, Meiosis, Spermatocytes, Oocytes, Meiotic prophase, Synaptonemal complex, DNA double-strand break repair

## Abstract

**Background:**

Sister chromatid cohesion mediated by the cohesin complex is essential for accurate chromosome segregation during mitosis and meiosis. Loading of cohesin onto chromosomes is dependent on another protein complex called kollerin, containing Nipbl/Scc2 and Mau2/Scc4. Nipbl is an evolutionarily conserved large protein whose haploinsufficiency in humans causes a developmental disorder called Cornelia de Lange syndrome. Although the function of Nipbl homologues for chromosome cohesion in meiotic cells of non-vertebrate models has been elucidated, Nipbl has not been characterized so far in mammalian spermatocytes or oocytes.

**Findings:**

Here we describe our analyses on the expression and localization of Nipbl in nuclei of mouse spermatocytes and oocytes at different stages of meiotic prophase. In both spermatocytes and oocytes we found that Nipbl is associated with the axial/lateral element of the synaptonemal complex (AE/LE) to which cohesin also localizes. Interestingly, Nipbl in spermatocytes, but not in oocytes, dissociates from the AE/LE at mid-pachytene stage coincident with completion of DNA double-strand break repair.

**Conclusions:**

Our data propose that cohesin loading activity is maintained during early stages of meiotic prophase in mammalian spermatocytes and oocytes.

## Background

Faithful chromosome segregation is ensured by cohesion between sister chromatids [1]. Cohesion is established during DNA replication and its loss in M-phase initiates chromosome segregation [2]. Cohesion is mediated by an evolutionarily conserved protein complex called cohesin, composed of two SMC (structural maintenance of chromosomes) proteins (Smc1, Smc3), an α-kleisin (Rad21/Scc1 or meiosis specific Rec8) and Scc3. Association of cohesin with chromosomes requires a binary protein complex called kollerin, consisting of a large, conserved protein called Nipbl (also known as Scc2) and a less well-conserved smaller protein called Mau-2 (or Scc4) [2]. Nipbl and its functional homologues have been characterized in different eukaryotic model organisms including budding and fission yeast, fungus *Coprinus*, nematodes, fruit flies, *Xenopus*, *Arabidopsis*, zebrafish and mammalian cells [3-12]. These studies have demonstrated that Nipbl is required for chromatin association of cohesin and thus proper chromosome segregation. Mutations in human *NIPBL* and genes encoding cohesin subunits cause a developmental disorder called Cornelia de Lange syndrome [13].

The majority of human aneuploidies are derived from mis-segregation of homologous chromosomes during meiosis I of oocytes [14]. To prevent this, sister chromatid cohesion must stably connect recombinant homologous chromosomes (manifested as bivalent chromosomes with chiasmata) at distal part of chromosome arms [15]. Indeed, mouse oocytes lacking the meiotic cohesin subunit *Smc1β* exhibit progressive loss of chiasmata and an increase in aneuploidy [16]. The discovery of maternal age-associated reduction of chromosome-bound cohesin further suggested that cohesin degeneration might promote age-related surge in aneuploidy [17-19]. Degeneration of cohesin during ageing might be due to lack of cohesin turnover [20,21]. Dysfunction of cohesin loading may also promote degeneration of cohesion. However, the cohesin loading factor Nipbl has not been studied thus far in mammalian meiotic cells. Nipbl homologues have been shown to localize to meiotic chromosomal axes in yeast, fruit flies, nematodes, *Arabidopsis* and *Coprinus*[8-12]. Nipbl mutations in some of these organisms are shown to cause meiotic cohesion defects and infertility, suggesting that Nipbl is responsible for cohesin loading also in meiotic cells. To gain insights into the function of Nipbl during mammalian meiosis, we investigated the localization of Nipbl in mouse spermatocytes and oocytes.

## Results and discussion

### Nipbl is expressed in mouse spermatocytes

**Figure 1:**
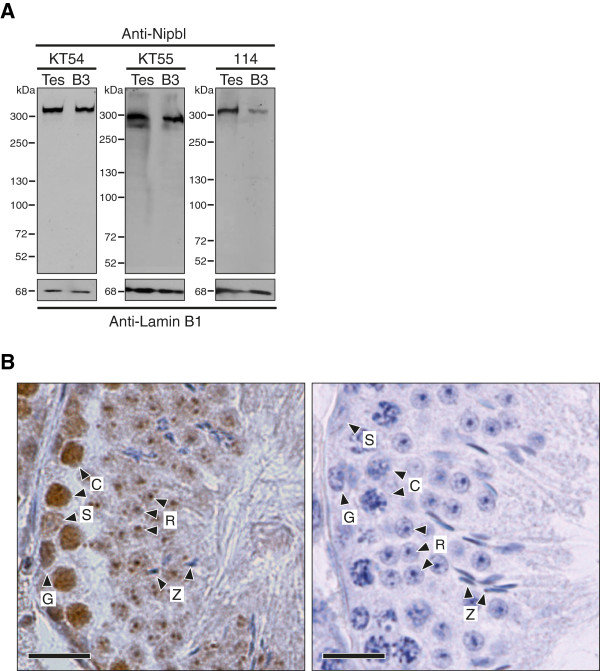
**Nipbl is expressed in mouse spermatocytes. (A)** Western blotting assays on total protein extracts from whole mouse testis (Tes) and mouse pre-B cell line (B3) [22] using antibodies against Nipbl. Anti-Lamin B1 was used as a loading control for each blot. **(B)** Immunohistochemistry on seminiferous tubule cross-sections from wild-type testis with (left) or without (right) the primary antibody KT55. Nuclei were counterstained with hematoxylin. G, spermatogonium; C, spermatocyte; R, round spermatid; Z, spermatozoon; S, Sertoli cell. Scale bar 200 μm.

Since Nipbl has not been studied in mammalian reproductive organs, we first examined expression of Nipbl in total protein extracts from mouse testes by Western blotting. Three antibodies against Nipbl have been successfully used for detecting human NIPBL; rat monoclonal antibodies KT54 and KT55, raised against peptide sequences of two splicing isoforms of human NIPBL (A; long form and B; short form) [7] and a rabbit polyclonal antibody 114 that recognizes both splicing variants [6]. The peptide sequences in NIPBL A and B used for raising these isoform-specific antibodies are conserved between human and mouse homologs. As shown in Figure 1A, all 3 antibodies detected one major protein that migrated at approximately 300 kDa in both testis and proliferating mouse pre-B cells, which is consistent with the deduced molecular weights of 315 and 304 kDa for mouse Nipbl A and B isoforms, respectively.

To identify cell types in the testis that express Nipbl, we performed immunohistochemistry on testis cross-sections using Nipbl antibodies. As expected, Nipbl was detected in mitotically proliferating male germ cells referred to as spermatogonia (marked as G in Figure 1B). We identified relatively high levels of Nipbl expression in meiotic spermatocytes (marked as C in Figure 1B). Anti-Nipbl signals were also detectable at chromocenters of post-meiotic round spermatids, but not in spermatozoa (marked as R and Z in Figure 1B, respectively). Accumulation of Nipbl was observed in quiescent Sertoli cells at slightly lower levels than in spermatogonia. Cohesin is known to be expressed in post-mitotic cells and recent studies have established that it has regulatory roles in gene expression [23-25]. In addition, cohesin is also required for DNA damage repair [26]. Induction of DNA double-strand breaks (DSBs) leads to cohesin reloading onto chromosomes, which is dependent on the activity of Nipbl [27-29]. Therefore, Nipbl may also be required for cohesin loading and turnover outside S-phase to support cohesin’s functions in quiescent cells. Expression of Nipbl in mouse spermatocytes suggests presence of the Nipbl-mediated cohesin loading activity during mammalian meiosis. In addition, these results indicate that these antibodies can be used reliably to specifically detect Nipbl protein.

### Localization of Nipbl in spermatocyte nuclei during meiotic prophase

**Figure 2:**
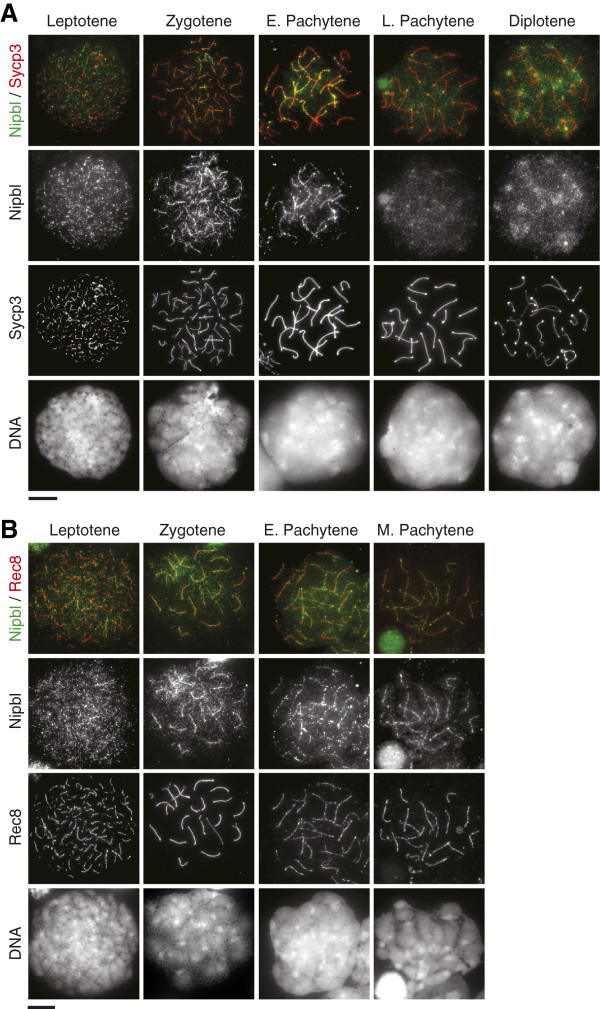
**Nipbl localization on spermatocyte chromosomes.** Immunofluorescence images of chromosome spreads stained with antibodies against Nipbl (green) and other marker proteins. No significant difference was observed in Nipbl signal patterns visualized by three Nipbl antibodies independently used for staining at each meiotic prophase stage (see the main text). Therefore these images represent localization of both Nipbl A and B isoforms. DNA was counterstained with DAPI. E, early; M, mid; L, late. Scale bars 10 μm. **(A)** Anti-Sycp3 (red) marks the AE/LE of synaptonemal complex. More than 20 nuclei were analyzed by each Nipbl antibody in each stage. **(B)** Anti-Rec8 (red) represents localization of cohesin that overlaps with the AE/LE during the stages shown. More than 15 nuclei were examined in each stage.

**Figure 3:**
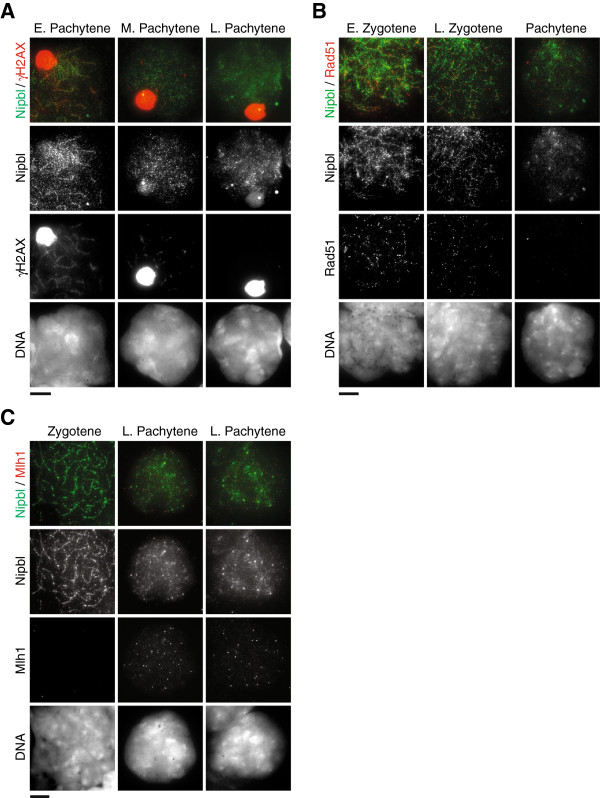
**Nipbl translocates during pachytene stage in spermatocytes.** Immunofluorescence images of chromosome spreads stained with antibodies against Nipbl (green) and other marker proteins. More than 5 nuclei were examined in each stage. DNA was counterstained with DAPI. E, early; M, mid; L, late. Scale bars 10 μm. **(A)** Anti-γH2AX (red) marks double strand breaks (DSBs). Note that anti-γH2AX highly accumulates at the sex body during the stages shown. **(B)** Rad51 (red) marks recombination intermediates. **(C)** Mlh1 (red) marks late recombination nodules.

To investigate Nipbl localization on spermatocyte chromosomes, we prepared testicular chromosome spreads and performed immunofluorescence examination using three Nipbl antibodies. We carefully compared signal patterns derived from each of three primary antibodies and found no significant difference amongst them (data not shown), suggesting that the Nipbl isoforms exhibit indistinguishable localization patterns. Co-staining with an antibody against Sycp3, a major component of the axial/lateral element of the synaptonemal complex (AE/LE), allowed us to stage meiotic prophase progression. Germ cells enter meiosis by replicating chromosomes, which is immediately followed by formation of Spo11-mediated DSBs during leptotene stage. Nipbl was detectable in leptotene nuclei as scattered foci throughout the nucleus (Figure 2A). These signals partially overlapped with anti-Rec8 signals (Figure 2B). Subsequently during zygotene stage, Nipbl accumulated at the AE/LE containing Sycp3 as well as cohesin (Figure 2A). The signals obtained with anti-Nipbl and anti-Rec8 at AE/LE were largely overlapping at the AE/LE (Figure 2B). The localization of Nipbl to AE/LE was maintained in early pachytene, but by late pachytene the anti-Nipbl signals at AE/LE became weaker (Figure 2A). Mid-pachytene is the stage where DSB is repaired, which coincides with disappearance of phosphorylated H2AX (γH2AX) (except on the sex chromosome pairs) and Rad51 signals from nuclei and appearance of recombination nodules marked by anti-Mlh1 [30]. To further investigate the timing of Nipbl relocalization, we performed co-staining of Nipbl with γH2AX, Rad51 or Mlh1 antibodies (Figure 3). We found that dissociation of Nipbl from the AE/LE takes place concomitantly with completion of DSB repair. In diplotene nuclei, enrichment of anti-Nipbl signals was detected at pericentromeric heterochromatin regions (Figure 2A). A previous study identified an interaction between Nipbl and heterochromatin binding protein 1 (HP1) [31], which suggests that Nipbl might translocate from the AE/LE to pericentromeric heterochromatin by interacting with HP1.

### Localization of Nipbl in fetal oocyte nuclei during meiotic prophase

**Figure 4:**
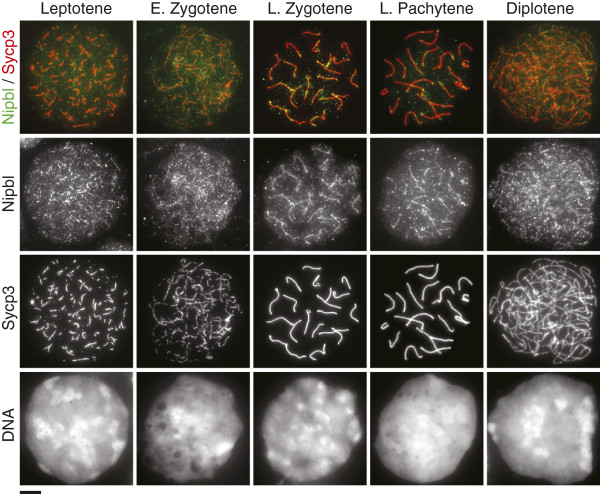
**Nipbl localization on oocyte chromosomes.** Immunofluorescence images of chromosome spreads during meiotic prophase stages in fetal ovaries stained with antibodies against Nipbl (green) and Sycp3 (red). Images for each stage represent both Nipbl A and B isoforms as no significant difference was identified in Nipbl signal patterns generated by all three antibodies (see the main text). More than 10 nuclei were examined in each stage. DNA was counterstained with DAPI. E, early; L, late. Scale bar 5 μm.

To investigate Nipbl localization on oocyte chromosomes during meiotic prophase, we performed immunofluorescence studies on chromosome spreads prepared from fetal ovaries (Figure 4). We used three Nipbl antibodies, but again found no significant difference in signal patterns at each stage (data not shown). Nipbl was detected in leptotene as foci throughout the nucleus and then accumulated on chromosome axes in zygotene, as observed in spermatocytes. We found that anti-Nipbl signals were enriched at chromosome axes throughout pachytene and diplotene stages, which is in contrast to the dissociation of Nipbl from chromosome axes upon DSB repair in spermatocytes.

## Conclusions

**Figure 5:**
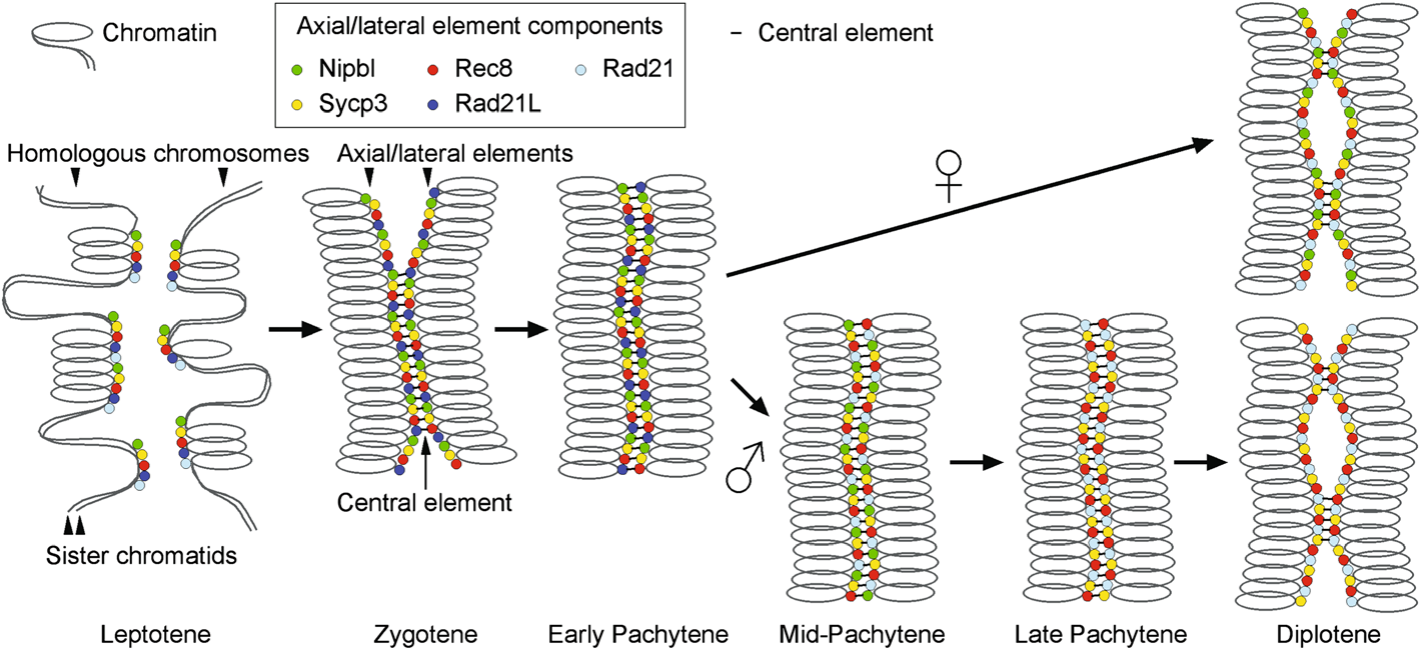
**Schematic for Nipbl localization during mammalian meiotic prophase.** Summary of our observations on Nipbl localization is illustrated together with the major component of AE/LE (Sycp3) and previously reported localization of the cohesin complexes containing different α-kleisin subunits (Rec8, Rad21 and Rad21L) during mouse meiotic prophase in spermatocytes and oocytes [30,32-36]. Note that we do not argue co-localization of each molecule in sub-regions of the AE/LE or molecule-to-molecule interactions.

In this report, we have described the sub-nuclear localization of Nipbl in different meiotic stages of mouse spermatocytes and oocytes (see Figure 5 for a schematic summary). Our cytological examinations show that Nipbl is bound to chromosomes in spermatocytes and oocytes soon after meiotic entry and accumulates at the AE/LE where it co-localizes with cohesin. This suggests that the cohesin loading activity is maintained during early stages of meiotic prophase in mammals. Interestingly, we found that Nipbl dissociates from the AE/LE upon DSB repair in pachytene spermatocytes. The novel member of meiosis-specific α-kleisin family called Rad21L has been identified recently that binds to the AE/LE until mid-pachytene and then dissociates from AE/LE with similar kinetics to Nipbl translocation [30,32]. With this timing the mitotic α-kleisin Rad21 becomes detectable on the AE/LE as if the cohesin complex containing Rad21 replaces that containing Rad21L. This temporal coincidence suggests that the cohesin loading activity may be necessary at the AE/LE until the loading of Rad21-containing cohesin complex. By contrast, the dissociation of Nipbl from the AE/LE during pachytene was not observed in oocytes. The functional significance for this sexual dimorphism remains unclear.

For our cytological analyses of Nipbl in mouse spermatocytes and oocytes during meiotic prophase we used three Nipbl antibodies. Two of these antibodies should discriminate two known isoforms (Nipbl A and B) and the third should detect both. However, we found no significant difference in signal patterns at each stage with these three antibodies, suggesting that Nipbl isoforms are indistinguishable in terms of localization during meiotic prophase.

Recent studies involving developmental stage-specific gene deletion or induction suggested the lack of *de novo* cohesin loading in meiotically arrested oocytes enclosed in ovarian follicles [20,21]. The nuclear phase of these arrested oocytes is called the dictyate stage, following diplotene, where the AE/LE has been disassembled. We did not detect significant levels of Nipbl on chromatin at this stage (data not shown), which is consistent with the observed lack of cohesin turnover. In the future, studies involving stage-specific*Nipbl* inactivation during mouse meiosis will be carried out to complement our cytological observations, which will further generate insights into dynamics and stability of the cohesin complex during mammalian gametogenesis.

## Methods

### Animals

All mice were housed in accordance with the Animals (Scientific Procedures) Act of 1986 and associated Codes of Practice and all experiments were performed under approved project licenses issued by the Home Office, UK. The mouse (*Mus musculus*) strains used in this study were bred in our facility and have mixed background of C57BL/6 J and 129/SV.

### Antibodies and detection

Antibodies were used as follows: rat monoclonal anti-Nipbl A (KT54, 2 F1) and anti-Nipbl B (KT55, 16H10) (Thermo Scientific) [7], rabbit anti-Nipbl (114, gift of J.-M. Peters) [6], rabbit anti-Rec8 [33], mouse monoclonal anti-Sycp3 (10G11, Abcam), rabbit anti-Sycp3 (15093, Abcam), rabbit anti-γH2AX (2577, Cell Signaling Technology), mouse monoclonal anti-Mlh1 (G168-15, BD Pharmingen), mouse monoclonal anti-Rad51 (14B4, Abcam). Appropriate secondary antibodies conjugated with Alexa Fluor fluorophore (Molecular Probes) were used. Whole cell extracts were prepared and subjected to Western blot analysis as described [37]. Histological preparation of testes was carried out as described [37] and immunohistochemistry was performed by Vectastain Elite ABC kit and ImmPACT DAB (Vector Labs) according to manufacturer’s instructions.

### Preparation and staining of chromosome spreads and microscopy

Preparation and staining of chromosome spreads from testes and fetal ovaries were performed as described previously [38,39]. Testes from 10 animals and ovaries from 6 fetuses were collected and processed for chromosome spreading. Wide-field epi-fluorescence microscopy was performed on a Leica DMI6000B equipped with an HCX PL APO 100×/1.40 Oil CS objective, dichroic filters (A4, L5, N3, TX2, Y5) and a Hamamatsu ORCA-ER CCD camera operated by the MetaMorph software (Molecular Devices). Bright-field microscopy was performed on an Olympus BX50 equipped with PlanApo (60×/1.40 and 40×/1.00) and PlanN (20×/0.40 and 10×/0.25) objectives and a Leica DFC295 color CCD camera operated by the LAS software.

## Abbreviations

AE/LE: Axial/lateral element of the synaptonemal complex; SMC: Structural maintenance of chromosomes; DSB: Double strand break.

## Competing interests

The authors declare that they have no competing interests.

## Authors’ contributions

KK and XF carried out the experiments, processed the data and evaluated data. NRK conceived the study, evaluated data and wrote the manuscript. All authors read and approved the final manuscript.

## Authors’ information

KK and XF are a PhD student and a postdoc, respectively, supervised by NRK.
